# The fate of systemically administrated allogeneic mesenchymal stem cells in mouse femoral fracture healing

**DOI:** 10.1186/s13287-015-0198-7

**Published:** 2015-10-26

**Authors:** Shuo Huang, Liangliang Xu, Yuxin Sun, Yifeng Zhang, Gang Li

**Affiliations:** Department of Orthopaedics & Traumatology, Faculty of Medicine, Room 904, 9/F, Li Ka Shing Institute of Health Institute, Prince of Wales Hospital, The Chinese University of Hong Kong, Shatin, 30-32 Ngan Shing Street, Shatin, NT, Hong Kong, SAR PR China; Stem Cells and Regenerative Medicine Laboratory, Li Ka Shing Institute of Health Sciences, The Chinese University of Hong Kong, Prince of Wales Hospital, 30-32 Ngan Shing Street, Shatin, NT, Hong Kong, PR China; Key Laboratory for Regenerative Medicine, Ministry of Education, School of Biomedical Sciences, Faculty of Medicine, The Chinese University of Hong Kong, Shatin NT, Hong Kong, SAR China; The CUHK-ACC Space Medicine Centre on Health Maintenance of Musculoskeletal System, The Chinese University of Hong Kong Shenzhen Research Institute, No. 10, 2nd Yuexing Road, South District, Hi-tech Park, Nanshan, 518057 Shenzhen, PR China; Lui Che Woo Institute of Innovative Medicine, Faculty of Medicine, The Chinese University of Hong Kong, 30-32 Ngan Shing Street, Shatin, NT, Hong Kong, SAR China

**Keywords:** Allogeneic mesenchymal stem cells (MSCs), systemic injection, local injection, fracture healing

## Abstract

**Introduction:**

The fate and whereabouts of the allogenic mesenchymal stem cells (MSCs) following their transplantation are not well understood. The present study investigated the fate of systemically administrated allogeneic MSCs in mouse fracture healing by using *in vivo* imaging and immunohistochemistry methods.

**Methods:**

Open femoral fracture with internal fixation was established in 30 FVB mice, which were assigned to three groups receiving phosphate-buffered saline (PBS) injection, MSC systemic injection, or MSC local injection. Luc-MSCs (5 × 10^5^) isolated from the luciferase transgenic mice with FVB background were injected at 4 days after fracture. All animals were terminated at 5 weeks after fracture; examinations included bioluminescence-based *in vivo* imaging, micro-computer tomography, mechanical testing, histology, immunohistochemistry, and double immunofluorescence staining.

**Results:**

The bioluminescence signals of the Luc-MSCs at the fracture site could be detected for 12–14 days following their injection in the Luc-MSC local injection group, whereas in the Luc-MSC systemic injection group, Luc-MSCs were initially trapped in lungs for about 8–9 days and then gradually redistributed to the fracture site. Bone mineral density, bone volume/tissue volume, ultimate load, and E-modulus in the MSC injection groups were significantly higher than those in the PBS group. Double immunostaining demonstrated that the MSC local injection group had more Luc-positive cells, and there was a higher apoptotic rate at the fracture site than the MSC systemic injection group. Both Luciferase-positive MSCs and osteoblasts were present in the callus in the MSC injection groups at 5 weeks after fracture, suggesting that some of allogenic Luc-MSCs contributed to the new bone formation. Only less than 3 % of injected Luc-MSCs remained at the fracture site in the MSC injection groups at 5 weeks following the fracture, and the rest of the injected Luc-MSCs disappeared.

**Conclusions:**

Our data showed that both systemic and local injection of allogeneic MSCs promoted fracture healing through enhancing biomechanical properties, bone content, and enlarged callus sizes. Immunohistochemistry confirmed that the injected MSCs are still present in the fracture site and can differentiate into osteoblasts to participate in fracture healing even at 5 weeks following the fracture. These findings provide useful information for the use of allogenic MSCs for cell therapy applications.

**Electronic supplementary material:**

The online version of this article (doi:10.1186/s13287-015-0198-7) contains supplementary material, which is available to authorized users.

## Introduction

Mesenchymal stem cells (MSCs), also known as multipotent mesenchymal stromal cells, have the innate ability to self-renew and differentiate into multiple cell types such as neurons, osteoblasts, cardiomyocytes, adipocytes, and chondrocytes when exposed to proper stimuli [1, 2], and MSCs applied locally have been used for treatments of various diseases [3–6]. Compelling evidence shows that MSC local application could repair tissue defects [7–9] and that MSCs are a promising cell source for tissue engineering [10, 11].

Although the use of autologous MSCs for cell therapy has been well established and accepted, the need of prolonged time for MSC culture to obtain therapeutic dose and the narrow time window for their optimal applications prohibit their wider applications. Thus, allogenic MSCs become an ideal alternative as they can be well prepared in advance, banked, and made readily available for use. However, the function and fate of allogeneic MSCs *in vivo* are still not well defined. Intravenous delivery of allogenic MSCs results in their specific migration to sites of injury and improves recovery in animal models of skin injury [12], stroke, and myocardial infarction [13–16]. In 2005, Shirley et al. reported that there was a systemic mobilization and recruitment of osteoblastic precursors to the fracture site via the peripheral circulation [17]. Caplan et al. also reported that MSCs delivered systemically via the circulatory system can home to target sites [18]. Taken together, allogenic MSCs applied locally and systemically could promote tissue (fracture) healing regeneration.

However, the function and fate of allogeneic MSCs *in vivo* are still not well defined. Some reports supported that MSCs mediate tissue and organ repair by replacing damaged cells [19, 20], and other studies suggest that allogeneic MSCs mainly play immune-modulatory roles *in vivo* [21–23]. Le Blanc et al. showed that MSCs could suppress the proliferation of both CD4^+^ and CD8^+^ T cells by upregulating the release of soluble factors such as interleukin-10 and prostaglandin E_2_ [24]. It was also reported that allogeneic MSCs encouraged repair through the production of trophic factors, cytokines, and antioxidants [25–27]. Kellie et al. also found that MSC treatment increased the tensile strength of wounds and increased production and deposition of collagens in the wound [28].

There are still issues of allogenic MSC application that need further investigation: What is the fate of the allogenic MSCs *in vivo*? How long they can function and survive *in vivo*? Is there any potential immunogenic effects caused by the allogenic MSCs? In the present study, we investigated the fate and effects of systemically administrated allogeneic MSCs versus local administration of allogenic MSCs in a mouse fracture healing model.

## Methods

### Chemicals

The chemicals used were all purchased from Sigma-Aldrich (St. Louis, MO, USA) except where specified.

### Animal details

Bone marrow-derived Luc-MSCs were isolated from 4-week-old female CMV-luc mice (FVB/N background; Xenogen Corporation, now part of Caliper Life Sciences, Hopkinton, MA, USA). Thirty FVB/N male mice (8 weeks old, body weight of 25–35 g) were used for fracture study. All mice were housed in a designated, government-approved animal facility at the Chinese University of Hong Kong in accordance with the Chinese University of Hong Kong animal experimental regulations, and all animal experiments were approved by the Animal Research Ethics Committee of the authors’ institution.

### Isolation and cultivation of mouse bone marrow-derived Luc-MSCs

The CMV-luc mouse was humanely terminated. Both tibias and femurs were excised, muscle and connective tissues were removed, and then bones were stored on ice in phosphate-buffered saline (PBS) with 1 % penicillin-streptomycin-neomycin (PSN). Under the laminar flow in a biological safety cabinet, two ends of bone were excised, and the marrow cavity was repeatedly flushed by 5 ml of alpha complete culture medium (with 15 % fetal bovine serum, 1 % PSN). All of the bone pieces were removed from the 100-mm culture dish by forceps and then incubated at 37 °C in a 5 % CO_2_ incubator for 5 days. The initial spindle-shaped cells appeared on day 3 under phase-contrast microscopy and then reached 70–90 % confluence within 2 days. Cells were trypsinized and re-plated by splitting at a ratio of 1:3 into new dishes. MSCs between passages 4–8 were used in this study. MSC markers CD44 and CD90, endothelial cell marker CD31, and hematopoietic marker CD45 were examined by flow cytometry in accordance with a previously published article [26]. The osteogenic and adipogenic differentiation abilities were characterized by Alizarin red and Oil red O staining after the corresponding inductions in accordance with our previously published article [29].

### Animal surgery

A mouse open transverse femoral fracture model with internal fixation was used. In brief, the mice were under general anesthesia and sterile conditions, and a lateral incision through shaved skin and fascia lata from the left knee to the greater trochanter was made. The plane between the vasti and hamstrings was then opened by blunt dissection to expose the femur. The exact centralization of the transverse osteotomy was made by hand saw. A small incision was opened at the knee level, and a hole was drilled at the inter-condylar notch by using a 23-gauge hypodermic needle. A custom-made stainless pin (diameter of 0.7 mm) was inserted into the right femoral bone marrow cavity at the knee level to fix the fracture. The incision was closed, and a radiography was then taken to confirm the fracture. After the surgery, the 30 mice were randomly assigned into one of three groups: the Luc-MSC local injection group (Loc group), the Luc-MSC systemic injection group (Sys group), or the PBS control group (PBS group); 10 mice were in each group.

### Cell injection

In this study, we used cardiac injection of MSCs directed by an ultrasound imaging system (Vevo 770; VisualSonics Inc., Toronto, ON, Canada) instead of intravenous injection, based on the following reasons: (1) the blood vessels in the mouse tail vein are thin; therefore, it is hard to be penetrated by the needle; moreover, the needle may break the vessel, resulting in cell leaking. (2) Our preliminary study showed that the cells injected through either the tail vein or the heart all ravel to the lungs within minutes of injection. There are more cells trapped in the lungs in the vein injection group comparing to the heart injection group.

Cells and PBS injection were carried out at 4 days after fracture. For the Luc-MSC systemic injection group, 5 × 10^5^ Luc-MSCs (in 100 μl of PBS) were injected into the left ventricle through heart puncture under the ultrasound imaging system; mice in the PBS control group were given 100 μl of PBS injection through heart puncture as above; for the local injection group, 5 × 10^5^ Luc-MSCs (in 100 μl of PBS) were directly injected into the fracture site.

### *In vivo* bioluminescent assays

After cell injection, five mice per Loc and Sys group were intra-peritoneally injected with D-Luciferin (15 mg/ml, 300 μl for a 30-g mouse). After 10 minutes, mice were subjected to the IVIS imaging examination, and the region of interest (ROI) was set in each image. The same parameter settings for IVIS imaging were used for all samples in this study: f number: 1, field of view: 22, binning factor: 16, luminescent exposure (seconds): 10. Mice were examined by IVIS imaging system every 2 days and thereafter until the signal disappeared. The rate of photons per second of ROI was calculated by IVIS software, the data were then analyzed by SPSS statistical software, and the intensity of the signal was expressed as percentages of photons per second of ROI. To minimize the variations between individuals for IVIS imaging detection, the IVIS 200 system was calibrated each day for the first use in accordance with the manufacturer’s manual, and all the IVIS *in vivo* imaging detection was carried out by two investigators who were blinded to the groups of animals tested (with animal coding unknown to the IVIS imaging machine operator).

### Micro-computer tomography examination

All 30 mice were terminated at 5 weeks after fracture. Eight mice per group were randomly chosen for micro-CT analysis. Right femurs of all 30 mice were excised; muscles, soft tissues, and the internal stainless pins were carefully removed. For image acquisition, 300 two-dimensional (2D) micro-tomographic slices with a 20-μm slice increment covering a total range of 6 mm were scanned by Scanco Medical μCT40 (Scanco Ltd., Brüttisellen, Switzerland). Two hundred fifty sequential slices of 2D CT images at 2.5 mm proximal and 2.5 mm distal to the fracture line were selected, and the contoured region including the cortical diaphyseal bone and endosteal callus was set in each image. A low-pass Gaussian filter (Gauss sigma = 0.8 and Gauss support = 1) was used to partly suppress the noise in the volumes. The high- and low-radio-opacity mineralized tissues were segmented by thresholding, and an appropriate threshold was determined from the grayscale CT images. Bone volume (BV), tissue volume (TV), BV/TV, and mean volumetric bone mineral density for each sample were recorded.

### Three-point bending mechanical testing

Tests were performed within 24 hours after excision at room temperature, and fractured femurs were tested to failure with a constant displacement rate of 4 mm/minute by a three-point bending device (H25KS; Hounsfield Test Equipment Ltd., Redhill, Surrey, UK). The femurs were loaded in the anterior-posterior direction with the span of the two support points set as 8 mm. The force loading point was set at the fracture site. After testing, the load-displacement curves of the femurs were generated by the built-in software (QMAT Professional Material testing software; Hounsfield Test Equipment Ltd.); ultimate load to failure, energy absorbed to failure (the area under the load-displacement curves, known as the toughness), and the modulus of elasticity (E-Modulus, the slope of the stress-strain curve, known as the tissue stiffness) [30] were recorded and analyzed by the software.

### Histology and immunohistochemistry

The fractured femurs were fixed in 4 % buffered formalin for 1 day and then decalcified with 9 % formic acid for 5-7 days. Attempts were made to standardize the sectioning at a mid-sagittal plane of each specimen by cutting the specimen in half (longitudinally in a sagittal plane) by using a slicing blade. Samples were subjected for tissue processing and then embedded in paraffin. Thin sections (5 μm) were cut on a Rotary Microtome (HM 355S; Thermo Fisher Scientific, Dreieich, Germany) along the long axis of each femur in the sagittal plane. Sections were mounted on the coated slides. Paraffin was removed by immersing the slides in Xylene 2 changes of 5 minutes at room temperature. Slides were then taken through graded ethanol and distilled water. For antigen retrieval, slides were immersed in 10 mM of citrate buffer at 60 °C for 20 minutes and then rinsed with PBS twice; the slides were then immersed in 3.0 % hydrogen peroxide in PBS for 5 minutes and rinsed twice with PBS for 5 minutes each.

### TUNEL assay and immunofluorescence staining

A peroxidase *in situ* apoptosis detection kit (catalog number S7100; EMD Millipore, Billerica, MA, USA) was used to detect the apoptosis level of injected Luc-MSCs. In brief, following the step above, slides were taken through equilibration buffer for 10 seconds and TDT enzyme in a humidified chamber at 37 °C for 1 hour. The slides were washed in PBS buffer for 10 minutes, then anti-digoxignenin conjugate was applied on the slides for 30 minutes, and this was followed by PBS washing and peroxidase substrate incubation for 6 minutes. The slides were finally counterstained in 0.5 % (wt/vol) methyl green for 10 minutes and washed in PBS. For detection of Luc-MSCs on the same slide, we performed immunostaining. Briefly, the counterstained slides were blocked by 5 % donkey serum in 1 % bovine serum albumin (BSA) for 20 minutes and then incubated with the goat anti-Luciferase antibody (1:300; Santa Cruz Biotechnology, Inc., Dallas, TX, USA) overnight at 4 °C. Slides were washed with PBS three times, incubated with the donkey anti-goat IgG-FITC (1:1000; Santa Cruz Biotechnology, Inc.) for 60 minutes at room temperature in the dark, and washed and mounted. The fluorescent cells were visualized by using a fluorescent microscope (Zeiss-spot; Carl Zeiss MicroImaging GmbH, Jena, Thuringia, Germany).

### Double immunofluorescence staining

We used two antibodies in one slide. In brief, slides were blocked by 5 % donkey serum in 1 % BSA for 20 minutes, incubated with the goat anti-Luciferase antibody (1:300; Santa Cruz Biotechnology, Inc.) and the rabbit anti-Osteocalcin antibody (1:300; Santa Cruz Biotechnology, Inc.) or the rabbit anti-Nestin antibody (1:300; Sigma-Aldrich) overnight at 4 °C, washed with PBS three times, and incubated with the donkey anti-goat IgG-FITC (1:1000; Santa Cruz Biotechnology, Inc.) and Cy3 donkey anti-rabbit IgG (1:1000; Life Technologies, Carlsbad, CA, USA) for 60 minutes at room temperature in the dark. Slides were washed with PBS three times, counterstained with 4′,6-diamidino-2-phenylindole (DAPI), mounted, and examined under fluorescent microscope.

### Quantitation of injected Luc-positive cells at the fracture site

The image with the maximum callus width in each sample was chosen for cell counting. Briefly, the image was opened by ImageJ version 1.48 (National Institute of Mental Health, Bethesda, MD, USA); when “Plugins/Analyze/Cell Counter” plugin is run, the Crosshair (mark and count) tool was used to manually count apoptotic and immunofluorescence-labeled cells. The cell counting result was generated by the software.

### Statistical analysis

All quantitative data were transferred to statistical spreadsheets and analyzed by a commercially available statistical program: SPSS version 16.0 (IBM Corporation, Armonk, NY, USA). One-way analysis of variance followed by *post hoc* test was used for comparison of mean values, and *P* values of less than 0.05 were considered statistically significant.

## Results

### The characterization of MSCs

The flow cytometry results confirmed that isolated Luc-MSCs were negative for hematopoietic marker CD45 (Additional file 1: Figure S1A) and endothelial cell marker CD31 (Additional file 1: Figure S1B) and homogenously positive for MSC markers CD44 and CD90 (Additional file 1: Figure S1C, D). In differentiation assays of Luc-MSCs, the Alizarin red staining demonstrated that mineralized nodules formed after 3 weeks of the osteogenic induction (Additional file 1: Figure S1E); intracellular Oil red O-stained lipid-rich vacuoles appeared after 2 weeks of the adipogenic induction (Additional file 1: Figure S1F).

### *In vivo* bioluminescent assays

**Fig. 1:**
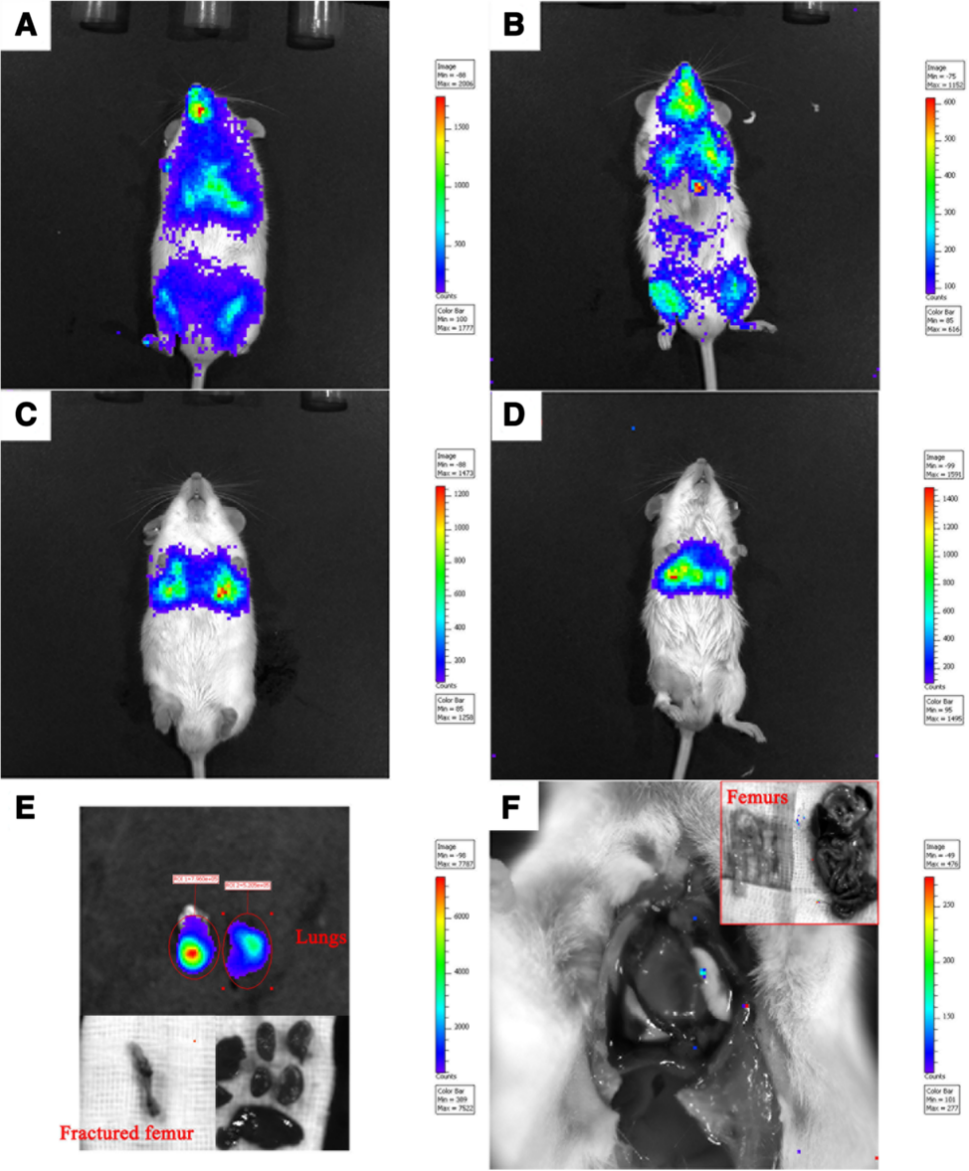
*In vivo* tracking systemically injected Luc-MSCs. **a**, **b** During the first hour following the injection, Luc-MSCs spread throughout the whole body via circulating system, and high concentrations of Luc-MSCs were observed in limbs, lungs, and oral cavity. **c**, **d** After about 1 hour following the injection, high concentrations of Luc-MSCs were observed only at the lung site, but no signal was observed at limbs, the oral cavity, and other parts of the body. **e** At day 4 following the injection, one mouse was terminated, and strong signals were observed at excised lungs, but no signal was observed on the excised fractured femur and other organs. **f** At day 11 following the injection, another mouse was terminated; no signal was observed throughout the whole body, including the lung site. *Luc-MSC* Luciferase labeled mesenchymal stem cell

**Fig. 2:**
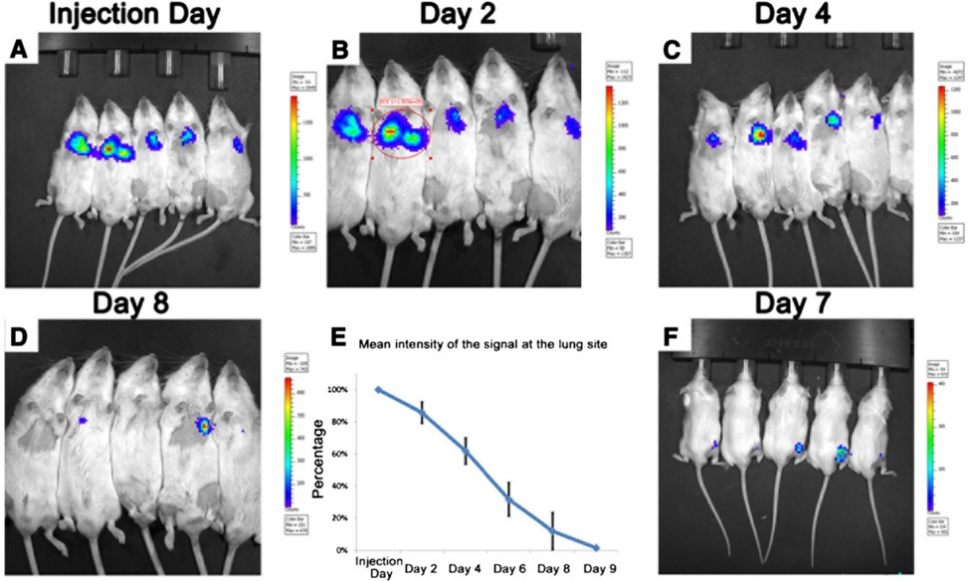
*In vivo* tracking Luc-MSCs in the systemic injection group. **a**–**d** The intensity of the signal at the lung site decreased gradually with time, and the signal lasted about 8–9 days following the systemic injection. **e** Quantitative analysis of the photons at the lung site also confirmed the above-mentioned phenomena. **f** At day 5–8 following the injection, very weak signals were observed at the fracture site and lasted 1–3 days. *Luc-MSC* Luciferase labeled mesenchymal stem cell

In the systemic injection group at the first hour following injection, Luc-MSCs spread throughout the body via circulating system, and higher concentrations of Luc-MSCs were seen in limbs, lungs, and oral cavity (Fig. 1a, b). After 1 hour following the injection, the Luc-MSCs aggregated in the lungs, and the signal at limbs, oral cavity, and other locations of the body was diminished (Fig. 1c, d). At day 4 following the injection, one mouse was terminated; the lungs, femurs, heart, liver, and kidney were excised and scanned by using the IVIS system; a strong signal was seen in the lungs, but none was seen at the fractured femur and other organs (Fig. 1e). At day 11 following the injection, another mouse was terminated, and there was no signal throughout the whole body, including the lung site (Fig. 1f). Figure 2a–d showed that the intensity of the signal at the lung site decreased gradually with time, and the detectable signals lasted about 8–9 days following the injection. Quantitative comparison of photons at different time points confirmed the continuous disappearance of the Luc-MSCs in the lungs (Fig. 2e). At day 5–8 following the injection, weak signals appeared at the fracture site (Fig. 2f) and lasted 1–3 days and then disappeared.

**Fig. 3:**
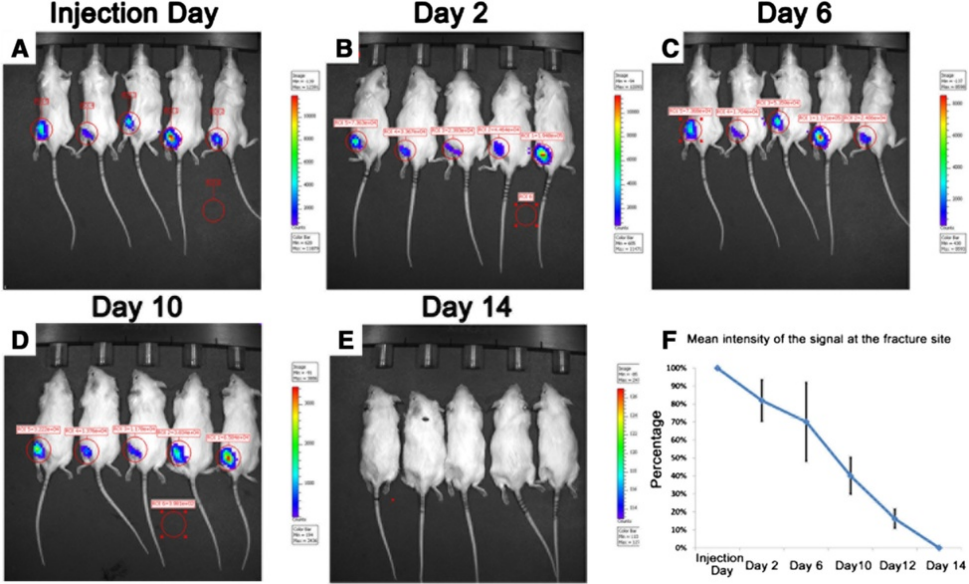
*In vivo* tracking Luc-MSCs in the local injection group. **a**–**e** The intensity of the signal at the fracture site decreased gradually with time, and the signal lasted about 12–14 days following the injection. **f** Quantitative analysis of the photons at the fracture site also confirmed the above-mentioned phenomena. *Luc-MSC* Luciferase labeled mesenchymal stem cell

In the local injection group, the intensity of the signal at the fracture site decreased gradually with time, and the signal lasted about 12–14 days following the injection (Fig. 3a, b, c, d, e) and that was confirmed by quantitative comparison of photons at different time points (Fig. 3f).

### Micro-CT analysis of the fractured bone

**Fig. 4:**
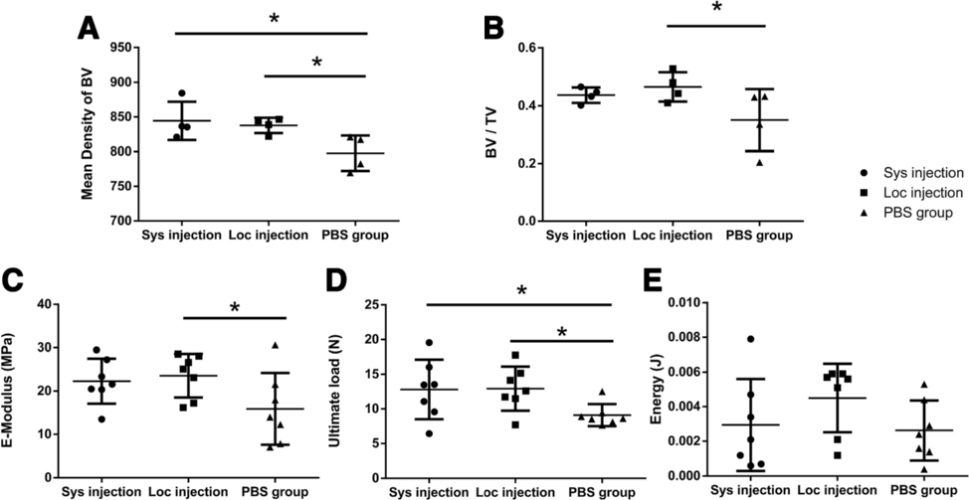
Micro-CT and three-point bending mechanical testing. **a** BV in the PBS control group was significantly lower than that of the MSC systemic injection group (*P* < 0.05) and the MSC local injection group (*P* < 0.05). **b** BV/TV in the MSC local injection group was significantly higher than that of the PBS control group (*P* < 0.05). **c** E-Modulus in the MSC local injection group was significantly higher than that of the PBS control group (*P* < 0.05). **d** Ultimate load to failure in the MSC systemic injection group was significantly higher than that of the PBS control group (*P* < 0.05), and the MSC local injection group was significantly higher than that of the PBS control group (*P* < 0.05). **e** Energy absorbed to failure did not show any significant difference among these three groups (*P* > 0.05). *BV* bone volume, *Luc-MSC* Luciferase labeled mesenchymal stem cell, *Micro-CT* micro-computed tomography, *MSC* mesenchymal stem cell, *PBS* phosphate-buffered saline, *TV* tissue volume

Quantitative analysis revealed that the mean density of bone volume in the PBS control group was significantly lower than that of the MSC systemic injection group (*P* < 0.05) and the MSC local injection group (*P* < 0.05), but there was no significant difference between the MSC systemic injection group and the MSC local injection group (Fig. 4a). The bone volume over tissue volume (BV/TV) in the MSC local injection group was significantly higher than that of the PBS control group (*P* < 0.05), but there was no significant difference between the MSC systemic injection group and the MSC local injection group (Fig. 4b).

### Three-point bending mechanical testing

E-Modulus (known as the tissue stiffness) in the MSC local injection group was significantly higher than that of the PBS control group (*P* < 0.05), but there was no significant difference between the MSC systemic injection group and the MSC local injection group (*P* > 0.05) and also no significant difference between the PBS control and the MSC systemic injection group (*P* > 0.05) (Fig. 4c).

Ultimate load to failure in the MSC systemic injection group was significantly higher than that of the PBS control group (*P* < 0.05), and the MSC local injection group was significantly higher than that of the PBS control group (*P* < 0.05), but there was no significant difference between the MSC systemic injection group and the MSC local injection group (*P* > 0.05) (Fig. 4d). Energy absorbed to failure (known as the toughness) did not show any significant difference among these three groups (*P* > 0.05) (Fig. 4e).

### Detection of apoptotic Luc-MSCs

**Fig. 5:**
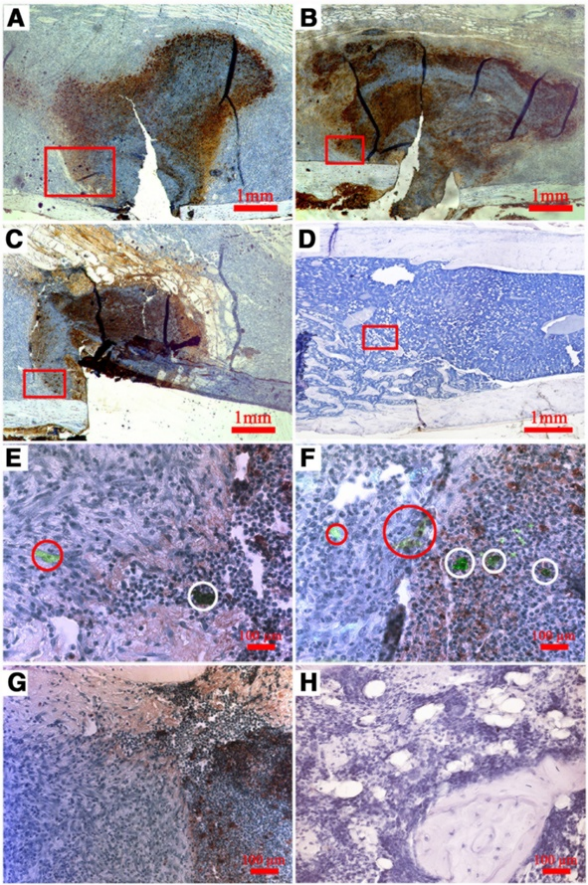
Illustration of apoptotic cells at the fracture site. A mass of apoptotic cells (brown cells) was observed around the fracture ends within the callus in the Luc-MSC systemic injection group (**a**), the local injection group (**b**), and the PBS control group (**c**) at 5 weeks following the fracture, but none was observed in the negative control (the normal femur) (**d**). **e**-**h** High-resolution images were, respectively, enlarged from the red outlined areas in a–d. Both living Luc-positive cells (light green cells within *red circles*) and apoptotic Luc-positive cells (dark green cells within *white circles*) were found in the MSC systemic injection group (**e**) and the MSC local injection group (**f**) at 5 weeks following the fracture, but no Luc-positive cells were found in the PBS control group (**g**) and the negative control (**h**). *Luc-MSC* Luciferase labeled mesenchymal stem cell, *MSC* mesenchymal stem cell, *PBS* phosphate-buffered saline

To examine the apoptosis level of injected Luc-MSCs, peroxidase staining and immunofluorescence staining were carried out on the same section. A mass of apoptotic cells (brown cells) was observed around the fracture sites within the callus in the Luc-MSC systemic injection group (Fig. 5a), the local injection group (Fig. 5b), and the PBS control group (Fig. 5c) at 5 weeks following the fracture, but none was observed in the negative control (the normal femur) (Fig. 5d).

High-resolution images were respectively enlarged from the red outlined areas in Fig. 5a, b, c, d (Fig. 5e, f, g, h). Both living Luc-positive cells (light green cells within red circles) and apoptotic Luc-positive cells (dark green cells within white circles) were found in the MSC systemic injection group (Fig. 5e) and the MSC local injection group (Fig. 5f) at 5 weeks following the fracture, but no Luc-positive cells were found in the PBS control group (Fig. 5g) and the negative control (Fig. 5h).

### Double immunofluorescence staining for Luc/Nestin and Luc/Osteocalcin

**Fig. 6:**
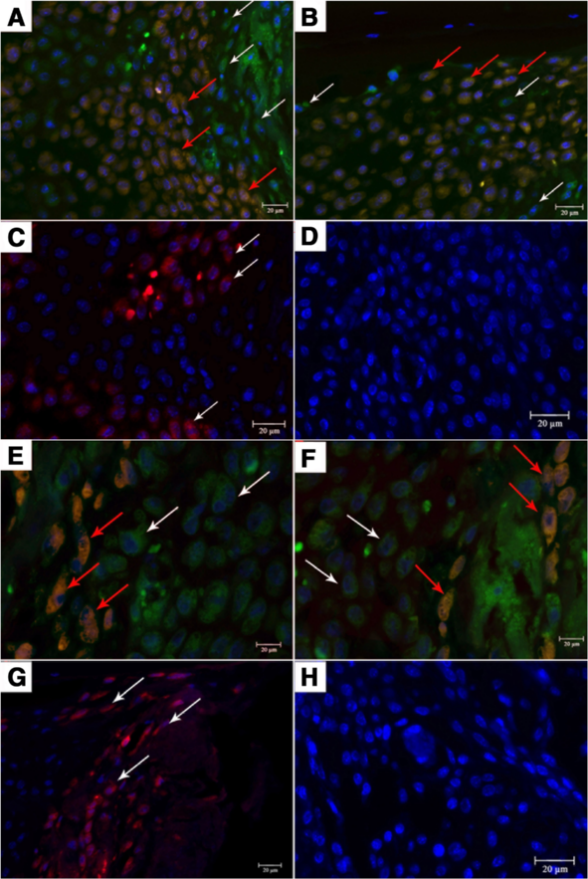
Double immunofluorescence staining for Luc/Nestin and Luc/Osteocalcin (DAPI for nucleuses). **a** In the Sys group, Luc^+^/Nestin^+^ cells (the red-yellow cells pointed out by *red arrows*), the systemically injected allogenic Luc-MSCs, had migrated to the fracture site. **b** In the Loc group, Luc^+^/Nestin^+^ cells (*red arrows*), the injected Luc-MSCs through local sites, remained at 5 weeks following the fracture. Luc^+^/Nestin^−^ cells (the green cells pointed out by *white arrows* in a, b) were observed, which meant that some injected Luc-MSCs had differentiated to other types of cells in both MSC injection groups. **c**, **d** Luciferase-positive cells were not observed in the PBS group (**c**) and the negative control (**d**), but Luc^−^/Nestin^+^ cells (*white arrows* in c), autogenetic MSCs, existed in the callus in the PBS control group. **e**, **f** Luc^+^/Osteocalcin^+^ cells (the red-yellow cells pointed out by *red arrows*) were observed both in the Sys group (**e**) and the Loc group (**f**), which meant that some injected Luc-MSCs had differentiated to osteoblasts. Luc^+^/Osteocalcin^−^ cells, the green cells (*white arrows*), were also found in MSC injection groups, which meant that some injected Luc cells remained at 5 weeks following the fracture. **g**, **h** Luciferase-positive cells were not observed in the PBS control group (**g**) and the negative control (**h**), but Luc^−^/Osteocalcin^+^ cells, autogenetic osteoblasts, were found in the callus. Scale bar: 20 μm. *DAPI* 4′,6-diamidino-2-phenylindole, *Luc-MSC* Luciferase labeled mesenchymal stem cell, *MSC* mesenchymal stem cell, *PBS* phosphate-buffered saline

To examine the co-distribution of luciferase (FITC immunolabeling) and Nestin (Cy3 immunolabeling), an MSC marker, double immunofluorescence staining was carried out on the same section. Luc^+^/Nestin^+^ cells, the red-yellow cells pointed out by red arrows in Fig. 6a, were observed in the MSC systemic injection group, which meant that some systemic injected allogenic Luc-MSCs had migrated to the fracture site via circulation system and still maintain their phenotype at 5 weeks following the fracture. Luc^+^/Nestin^+^ cells (red arrows, Fig. 6b) were observed in the MSC local injection group, which meant that some injected Luc-MSCs through local sites remained at 5 weeks following the fracture. Luc^+^/Nestin^−^ cells, the green cells pointed out by white arrows in Fig. 6a, b, were also observed, which meant that some injected Luc-MSCs had differentiated into other types of cells in both MSC injection groups. Luciferase-positive cells were not observed in the PBS control group (Fig. 6c) and the negative control (without the primary antibodies) (Fig. 6d), but Luc^−^/Nestin^+^ cells were found in the PBS control group (white arrows in Fig. 6c), which meant that autogenetic MSCs existed at the fracture site in the PBS control group.

Double immunofluorescence staining was also carried out to examine the co-distribution of luciferase (FITC immunolabeling) and Osteocalcin (Cy3 immunolabeling), secreted solely by osteoblasts on the same section. Luc^+^/Osteocalcin^+^ cells, the red-yellow cells pointed out by red arrows, were observed in both the MSC systemic injection group (Fig. 6e) and the MSC local injection group (Fig. 6f) at 5 weeks following the fracture, which meant that some injected Luc-MSCs had differentiated into osteoblasts at 5 weeks following the fracture and contributed to the bone formation. Luc^+^/Osteocalcin^−^ cells, the green cells pointed out by white arrows in Fig. 6e, f, were also found in both systemic and local injection groups, which confirmed that some injected Luc-positive cells remained at the fracture sites at 5 weeks following the fracture. Luc-positive cells were not observed in the PBS control group (Fig. 6g) and the negative control (without primary antibodies) (Fig. 6h), but Luc^−^/Osteocalcin^+^ cells were found in the PBS control group (white arrows in Fig. 6g), which meant that autogenetic osteoblasts existed at the fracture site at 5 weeks following the fracture and contributed to the bone formation.

### The number of injected Luc-positive cells at the fracture site

**Fig. 7:**
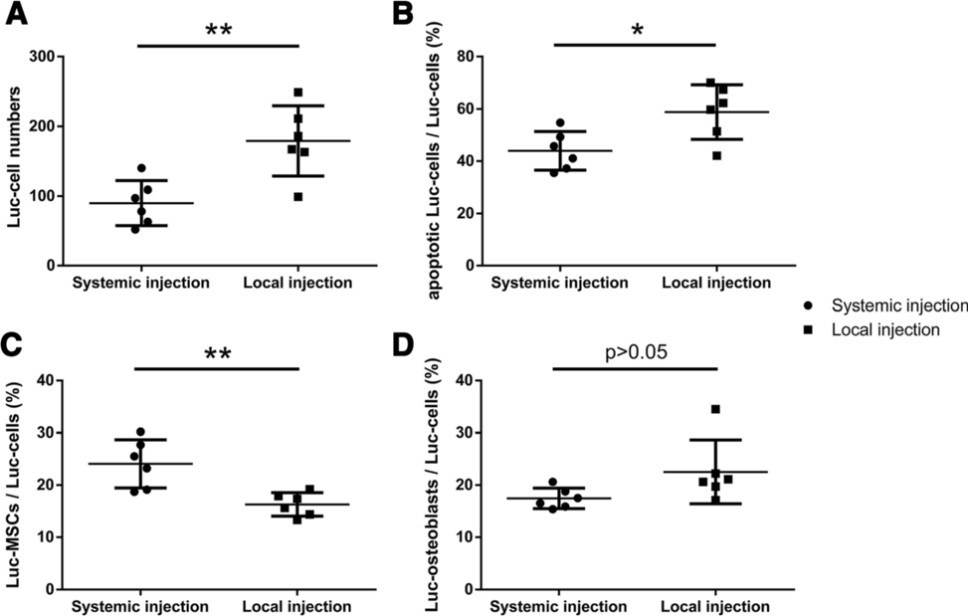
Comparison of the number of the injected Luc-positive cells at the fracture site at 5 weeks following fracture. **a** The number of Luc-positive cells in the callus in the MSC systemic injection group was significantly lower than that of the MSC local injection group (*P* < 0.05). **b** The proportion of apoptotic Luc-positive cells in Luc-positive cells in the MSC systemic injection group was significantly lower than that in the MSC local injection group (*P* < 0.05). **c** The proportion of Luc-MSCs in Luc-positive cells in the MSC systemic injection group was significantly higher than that of the MSC local injection group (*P* < 0.01). **d** The proportion of Luc-osteoblasts in Luc-positive cells did not show significant difference between the MSC systemic injection group and the MSC local injection group (*P* > 0.05). *Luc-MSC* Luciferase labeled mesenchymal stem cell, *MSC* mesenchymal stem cell

Quantitative analysis revealed that the number of Luc-positive cells in the callus in the MSC systemic injection group was significantly lower than that of the MSC local injection group (*P* < 0.05) (Fig. 7a). The proportion of apoptotic Luc-positive cells in Luc-positive cells in the MSC systemic injection group was significantly lower than that in the MSC local injection group (*P* < 0.05) (Fig. 7b). The proportion of Luc-MSCs in Luc-positive cells in the MSC systemic injection group was significantly higher than that of the MSC local injection group (*P* < 0.01) (Fig. 7c). The proportion of Luc-osteoblasts in Luc-positive cells did not show a significant difference between the MSC systemic injection group and the MSC local injection group (*P* > 0.05) (Fig. 7d).

### The minimal Luc cells that IVIS *in vivo* imaging system could detect *in vivo* in mice

**Fig. 8:**
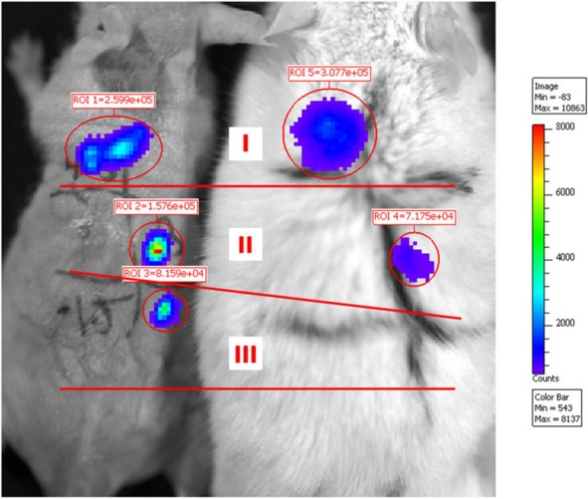
*In vivo* tracking injected Luc-positive cells by IVIS 200. Luc-MSCs (1.5 × 10^4^, 3 × 10^4^, and 4.5 × 10^4^) with luciferin were subcutaneously injected into three different areas of the nude and FVB (with hairs) mouse (area I: 4.5 × 10^4^ cells, II: 3 × 10^4^ cells, III: 1.5 × 10^4^ cells). Luc cells (1.5 × 10^4^) in the FVB mouse cannot be found by IVIS 200. *Luc-MSC* Luciferase labeled mesenchymal stem cell, *MSC* mesenchymal stem cell

IVIS *in vivo* imaging systems, based on the principle of bioluminescence, are widely used for *in vivo* tracking and monitoring of Luciferase-expressing cells in recipient animals [29, 31]. However, it was reported that Luciferase expression was observed to decrease with time, most likely the result of tissue rejection [32]. The light signal detected on the surface of the mouse also depends on the depth of the injected cells below the tissue surface and the number of Luc cells. To test the sensitivity and the accuracy of the IVIS imaging system, we subcutaneously injected 1.5 × 10^4^, 3 × 10^4^, and 4.5 × 10^4^ Luc-MSCs with luciferin, respectively, into three different areas of the nude mouse and the FVB mouse (with hair) (area I: 4.5 × 10^4^ cells, II: 3 × 10^4^ cells, III: 1.5 × 10^4^ cells; Fig. 8), and then we monitored both mice by using IVIS. The results showed that area III in the FVB mouse did not show any signal under IVIS but did in the nude mouse (Fig. 8). Concerning the foregoing results, in the systemic injection group, the signal at the fracture site was found only at day 5–8 following the injection by IVIS; in the local injection group, the signal at the fracture site lasted only 12–14 days; however, Luc-positive cells could be found by immunofluorescence staining at 5 weeks following the fracture in both MSC injection groups; therefore, in the present studies, we concluded that the IVIS 200 *in vivo* imaging system was sensitive to detect the number of the Luc cells above 1.5 × 10^4^*in vivo* in mice.

## Discussion

### Systemic and local injection of allogenic MSCs promoted fracture healing equally in this study

Systemic injection is more appropriate for treatments of systemic diseases such as osteoporosis, injuries involving deeper sites, and poly-trauma; and local injection is simpler for single and superficial injures such as skin wound or burns [33]. The present study has demonstrated that both systemic and local administration of allogeneic MSCs promoted fracture repair significantly, and obvious immune responses in this study were not observed in either allogeneic MSC systemic or local administration. Therefore, the use of allogeneic MSCs either systemically or locally is effective in the mouse fracture model.

The explanations for the equal effects in promoting fracture healing in both systemic and local injection groups are that (1) the limited sample size used in this study may not verify the small difference, (2) systemically and locally injected cells may contribute to the fracture healing in different ways, (3) high dose of injected cells ensured the equal effects, and (4) our data showed that the number of systemically injected Luc cells at the fracture site were significantly less than those of locally injected Luc cells because of the blood barriers in the lungs; however, the proportion of apoptotic Luc-positive cells in Luc-positive cells in the MSC systemic injection group was also significantly lower than that in the MSC local injection group, which may be credited to the low pH, hypoxia, inflammation, and infection in the local environment.

The *in vivo* imaging is a very useful technique and an invaluable tool to examine the living cells *in vivo*, and at present the IVIS system is the best research tool for tracing luciferase-labeled cells *in vivo* in small animals (mice). However, there is a limitation of IVIS system, and in the present study we found that the IVIS 200 system was not sensitive enough to detect the number of the Luc cells below 1.5 × 10^4^*in vivo* in mice; hence, the use of immunohistochemistry methods to detect the Luc-positive cells will be necessary to confirm the whereabouts of the Luc-positive cells when the cells numbers are expected to be lower.

### The fate of engrafted MSCs

*In vivo* imaging data showed the following: (1) For heart injection, MSCs spread throughout the whole body via the circulating system in the early hours after the injection and aggregated into lungs, gradually decreased with time, and disappeared after about 8–9 days. Injected MSCs could be found at the fracture site at day 5–8 following the injection. (2) For local injection, engrafted MSCs at the fracture site decreased gradually with time, and the Luc cells disappeared at about 12–14 days following the injection.

Our double Immunofluorescence staining results showed that both systemically and locally injected Luc-MSCs were observed in the callus even at 5 weeks following the fracture, and some of them had differentiated into osteoblasts and directly contribute to the bone formation. However, as we discussed above, IVIS 200 was not sensitive to the number of the Luc cells below 1.5 × 10^4^*in vivo* in the FVB mouse, which meant that less than 3 % of injected Luc cells (1.5 × 10^4^/5 × 10^5^) remained at the fracture site in the MSC injection groups at 5 weeks following the fracture, and about half of these cells went apoptosis in the callus. Our preliminary studies also showed that the systemically injected Luc-MSCs were not found in the heart, liver, kidney, and lungs by using immunofluorescence staining at 5 weeks following the fracture, which meant that almost 98.5 % of the injected Luc cells were dead after 1 month following the transplantation.

## Conclusions

In this study, we tracked and estimated the systemically and locally injected MSCs in both lungs and fracture sites by using *in vivo* imaging system, which may give guidance for the clinical transplantation of MSCs. Our data also showed that both systemic and local administration of allogeneic bone marrow-derived MSCs promoted mouse fracture healing and enhanced callus formation and their mechanical properties. However, we have not understood the exact cellular and molecular mechanisms behind the systemic allogeneic MSC administration in promoting fracture repair, and they still need further investigations.

## Additional file

Additional file 1: Figure S1. Cell surface markers and differentiation capacities of mouse MSCs. (**A**) Flow cytometry analysis results confirmed that isolated BM-MSCs were negative for hematopoietic marker CD45. (**B**) Cells were negative for endothelial cell marker CD31. (**C**, **D**) Cells were positive for MSC markers CD44 and CD90. (**E**) The Alizarin red staining demonstrated that mineralized nodules formed after 4 weeks of the osteogenic induction. (**F**) Intracellular Oil red O-stained lipid-rich vacuoles appeared after 2 weeks of the adipogenic induction. *BM-MSC* bone marrow-derived mesenchymal stem cell, *MSC* mesenchymal stem cell. (TIFF 468 kb)

## Abbreviations

BSA: Bovine serum albumin
BV: Bone volume
FITC: Fluorescein isothiocyanate
Luc-MSC: Luciferase labeled mesenchymal stem cell
Micro-CT: Micro-computed tomography
MSC: Mesenchymal stem cell
PBS: Phosphate-buffered saline
TV: Tissue volume
